# Tracking the Growth of Superparamagnetic Nanoparticles with an *In-Situ* Magnetic Particle Spectrometer (INSPECT)

**DOI:** 10.1038/s41598-019-46882-6

**Published:** 2019-07-22

**Authors:** Ankit Malhotra, Anselm von Gladiss, André Behrends, Thomas Friedrich, Alexander Neumann, Thorsten M. Buzug, Kerstin Lüdtke-Buzug

**Affiliations:** Institute of Medical Engineering, University of Lübeck, Ratzeburger Allee 160, Lübeck, Germany

**Keywords:** Nanoparticle synthesis, Biomedical engineering, Electrical and electronic engineering, Characterization and analytical techniques, Characterization and analytical techniques

## Abstract

Magnetic Particle Spectroscopy (MPS) is a measurement technique to determine the magnetic properties of superparamagnetic iron oxide nanoparticles (SPIONs) in an oscillating magnetic field as applied in Magnetic Particle Imaging (MPI). State of the art MPS devices are solely capable of measuring the magnetization response of the SPIONs to an oscillatory magnetic excitation retrospectively, i.e. after the synthesis process. In this contribution, a novel *in-situ* magnetic particle spectrometer (INSPECT) is presented, which can be used to monitor the entire synthesis process from particle genesis via growth to the stable colloidal suspension of the nanoparticles in real time. The device is suitable for the use in a biochemistry environment. It has a chamber size of 72 mm such that a 100 ml reaction flask can be used for synthesis. For an alkaline-based precipitation, the change of magnetic properties of SPIONs during the nucleation and growth phase of the synthesis is demonstrated. The device is able to record the changes in the amplitude and phase spectra, and, in turn, the hysteresis. Hence, it is a powerful tool for an in-depth understanding of the nanoparticle formation dynamics during the synthesis process.

## Introduction

Magnetic Particle Spectroscopy (MPS) is a technique to characterize superparamagnetic iron oxide nanoparticles (SPIONs) for their possible performance in magnetic particle imaging (MPI). MPI is a tomographic imaging modality measuring the spatial distribution of the SPIONs^1,2^. It is a quantitative imaging technique, which provides high sensitivity and can provide submillimeter spatial resolution^3^. The hardware realization for MPI is possible in a number of ways and is illustrated by Buzug *et al*.^4^. The properties of the magnetic particles strongly influence their usage for MPI or other applications. The quality of MPI images, for example, strongly depends on the anisotropy and size distribution of the SPIONs used as tracer material^3^. The nanoparticles used for the first experiments in MPI were predominately developed for magnetic resonance imaging (MRI) and are of limited use in the excitation fields of an MPI scanner. Moreover, magnetic nanoparticles have also been used in magnetic fluid hyperthermia (MFH)^5^. In MFH the nanoparticles serve as a medium for deposition of energy to a specific area using hysteresis losses^6^. Therefore, the hysteresis of the particles plays a critical role for most applications. Furthermore, SPIONs can be used for drug delivery^7^. In general, one can state that different fields of application require individually tailored nanoparticles. Actually, there is immense research to produce SPIONs, which are specifically tailored for the use in MPI as well as for MFH-based therapy^8,9^. These new types of SPIONs should have defined magnetic properties (e.g. saturation magnetization, steepness of magnetization, anisotropy) as well as geometrical properties (core diameter, hydrodynamic diameter distribution in suspension) to show optimal results in MPI. There are a lot of commercially available particles which have been characterized for MPI and compared according to their magnetic properties with the help of an MPS^10^. For example, nanoparticles with a core diameter of 20 nm and hydrodynamic diameter of 30 nm showed the best MPI performance so far^11^.

For MFH the hysteresis curve area should be large enough to deposit the required energy to cause cell ablation. For example, the larger the size of the nanoparticles, the steeper is the rise in the magnetization curve leading to the measurement of higher harmonics^12^. Due to this, the particles require more energy to get magnetized, which leads to the requirement of a higher field strength. Furthermore, the Brownian and Néel relaxation time increase with increasing size^13,14^.

In addition to the magnetic properties, the geometrical properties such as size and shape of the nanoparticles are of major importance for the biodistribution and biocompatibility in biomedical applications^15–17^. Moreover, the pH of the solution affects the susceptibility of the SPION solution as reported by Lucchini *et al*.^18^. These properties cannot be measured with the help of an MPS device, but they play an essential role in the imaging process as well as in therapeutic applications. It is worth mentioning that the change in temperature has a direct effect on the response of SPIONs in an MPS. This is due to the fact that the Brownian relaxation time (*τ*_*B*_) and the Néel relaxation time (*τ*_*N*_) are dependent on the thermal energy. Moreover, heating leads to a change in viscosity which also affects the Brownian relaxation time (*τ*_*B*_)^19,20^.

There are a variety of techniques for measuring the magnetic properties of SPIONs such as vibrating sample magnetometry that measures the static magnetization curve and the AC magnetometry, which measures the magnetic susceptibility. Unfortunately, none of these are able to measure the spectral magnetic moment at the desired frequency and field strength used in an MPI scanner. On the other hand, there has been an immense progress in the development of MPS devices as, for instance, one-dimensional MPS without offset and with various excitation frequencies, two-dimensional Lissajous MPS, arbitrary-waveform spectrometer and many more^21–23^, developed for different MPI associated applications. Moreover, there are also new approaches to characterize the SPIONs such as a frequency-mixing method based on search coils, which measure the phase lag of the magnetic moment compared to the drive field, giving more precise information regarding saturation magnetization and average hydrodynamic size^24^. The measurements from MPS devices usually consist of amplitude and phase spectra. The amplitude spectra contain both the even and odd harmonics, odd harmonics are attributed to the particle signal and even harmonics are caused due to the presence of a DC offset magnetic field. These MPS devices are able to provide information regarding physical and geometrical particle properties after synthesis completion. Therefore, the synthesis process is largely unobserved and the nanoparticles produced are characterized after the completion of the synthesis process giving no information regarding the nucleation and growth of the SPIONs. Other methods such as X-ray scattering or transmission electron microscopy are being used for real-time monitoring of nanoparticles growth^25,26^, but these devices have a very tedious setup and cannot be used in a normal chemistry laboratory. Real-time transmission electron microscopy is a very advanced tool and provides optical and structural information in the nanometer to sub-Angstrom scale, but requires special *in-situ* chambers for carrying out the chemical reactions^27–29^. X-ray diffraction techniques do not provide any optical details but also require special experimentation chambers for observing different samples at higher temperatures and pressures^30,31^. Moreover, X-ray diffraction is very useful in studying mechanochemical reactions^32,33^ Contrarily, *in-situ* Magnetic Particle Spectrometer (INSPECT) does not require any special sample chambers or experimentation setups for conducting syntheses. Moreover, INSPECT is a low-cost device with low instrumentation complexity and does not require user expertise in comparison to X-ray diffraction and TEM. INSPECT directly gives access to the change in the magnetic moment during the synthesis, a parameter which is important for magnetic imaging (e.g. MPI) and magnetic thermo-therapeutic scenarios. However, one limitation of INSPECT is that it relies on the magnetic properties of the SPIONs and hence cannot be used with other nanostructures.

Synthesis research is a time-consuming and difficult task because there has been no real-time control of the synthesis process so far. The control of the synthesis parameters has been carried out in the case of practical blindness, as there was no insight into the current synthesis situation. The aim of this research is to develop a spectrometer for the real-time monitoring of the changes in the magnetic properties of the SPIONs during synthesis. To achieve this, a novel *in-situ* Magnetic Particle Spectrometer (INSPECT) is introduced. INSPECT measures the magnetization response of the tracer material during a sinusoidal magnetic excitation *in-situ*. The INSPECT hardware is based on the same principles as typical MPS^21^, but contains less hardware components and is adapted for usage in a chemistry laboratory. In Theory, a brief description of MPS related magnetism physics and the particle nucleation and growth process are given. The dedicated hardware and software as well as the laboratory setup and basic chemical reactions are described in Materials and Methods. In Results and Discussion, initial results obtained by performing a synthesis process within the INSPECT reaction chamber are shown. A brief conclusion concludes this contribution.

## Theory

This section deals with the basics of field strength calculations in a solenoid and the induced signal in the receiving coil. Moreover, a brief introduction to the chemical nanoparticle synthesis process including nucleation and particle growth is given.

### Magnetic field strength

The magnetic field strength produced by a solenoid can be determined with the Biot-Savart law and Ampere’s law. In particular, the axial magnetic flux density near the center of the solenoid with length *L*, number of windings *N*, the applied current *I*, *μ*_0_ is the permeability of vacuum and *n* the density of turns (turns per meter length of the solenoid) can be determined by

1 $$B=\frac{{\mu }_{0}NI}{L}={\mu }_{0}nI,n=\frac{N}{L}$$

### Induced signal

The induced voltage *u*(*t*) in a receiving coil due to a temporal change in the particle magnetization ***M***(***x***, *t*) can be calculated by using the reciprocity principle for magnetic recording^34^

2 $$u(t)=-{\mu }_{0}\mathop{\int }\limits_{v}\,{p}_{0}{\boldsymbol{(}}x{\boldsymbol{)}}\frac{d}{dt}M(x,t)\,dV$$

where *V* is the sample volume, ***p***_***0***_ is the spatial sensitivity of the coil, and ***x*** denotes the x-direction of the magnetic field. For simplicity, only a homogeneous generated magnetic field is considered. The average coil sensitivity ***p***_***0***_ is given as ${p}_{0}={\int }_{v}{{\boldsymbol{p}}}_{{\bf{0}}}({\boldsymbol{x}})\cdot {e}_{x}\,dV$, where, ***p***_**0**_(***x***) = ***H***_***r***_(***x***)/*i*_0_ and ***H***_***r***_(***x***) is the magnetic field generated by the receiving coil due to current *i*_0_. Using the above equation, the frequency spectrum of the magnetization $\hat{m}(f)$ is defined as^21^

3 $$\hat{m}(f)=\frac{j}{2\pi f}\frac{1}{{\mu }_{0}{p}_{0}}{\int }_{0}^{T}\,u(t){e}^{-j2\pi ft}\,dt$$

with *f* the frequency, *T* = 1/*f*_0_ and *f*_0_ the fundamental frequency.

### Particle theory

The non-linear magnetization curve of a SPION is typically modeled by the Langevin theory of paramagnetism^35^

4 $${M}_{D}(\xi )={m}_{s}c(\coth \,(\xi )-\frac{1}{\xi }),$$

where ${m}_{s}=\frac{1}{6}\pi {D}^{3}{M}_{s}$ is the magnetic moment at saturation of a single particle and *c* is the concentration of the particles. The Langevin parameter *ξ* can be defined as $\frac{{m}_{s}{\mu }_{0}H(t)}{{k}_{B}T}$ where *k*_*B*_ is the Boltzmann constant and *T* is the absolute temperature. The Langevin model is rather simple and neglects the size distribution of particles. Therefore, there are different saturation magnetizations *M*_*s*_ of the particles in suspension. Hence, the magnetizations *M*_*D*_(*t*) have to be averaged. The resulting bulk magnetization $\tilde{M}(t)$ is defined as

5 $$\tilde{M}(t)={\int }_{0}^{\infty }\,\rho (D){M}_{D}(t)dD$$

where *ρ*(*D*) is the probability density function of the particle size distribution. Using the bulk magnetization $\tilde{M}(t)$, the induced voltage *u*(*t*) and the magnetic moment spectrum $\hat{m}(f)$ of the particle ensemble can be calculated^21^. But the Langevin model just provides a static model for the magnetic response of SPIONs. The Debye model corresponds to the magnetic response of SPIONs to a weak excitation field of frequency *ω*. According to the model, the real part of the magnetic susceptibility *χ*′(*ω*) monotonically decreases with the increase in frequency. Therefore, by using the value of *ω* it is possible to ascertain the mean value of Brownian relaxation time (*τ*_*B*_) more precisely^36–38^.

### Fundamentals of nanoparticle nucleation and growth

Nucleation and growth of nanoparticles are a very complex multi-step process. Here a simplified model for the nucleation and growth is explained. The most important factor responsible for the nucleation and growth is a change in Gibbs free energy Δ*G*_*V*_, which depends on the concentration of the solute and can be described as

6 $${\rm{\Delta }}{G}_{V}=\frac{{k}_{b}T}{{\rm{\Omega }}}\,\mathrm{ln}\,\frac{c}{{c}_{0}}=-\,\frac{{k}_{b}T}{{\rm{\Omega }}}\,\mathrm{ln}\,(1+\sigma ),$$

where *c* is the concentration of the solute, *c*_0_ is the equilibrium concentration or solubility, Ω is the atomic volume of the solid phase and *σ* is the supersaturation (when the solution contains higher volume of the solute which can be dissolved in a solvent) defined by (*c* − *c*_0_)/*c*_0_. If there is no supersaturation (*σ* = 0), then no nucleation will happen and hence no particle growth. For the stability of the nuclei, it is important that they reach a critical size or they would dissolve back into the solution causing a reduction of the Gibbs free energy^39^. After the attainment of a critical radius the growth process will continue without any barrier. To achieve monodispersity, it is important that no new nuclei are formed after the initiation of the growth process. Therefore, the ideal condition for synthesizing monodisperse SPIONs is the formation of the nuclei in a very short time interval subsequently leading to a coherent growth process, which is highly dependent on the kinetics and change of the surface energy and can be controlled by changing different parameters of the chemical reaction such as temperature and the amount of solutes and solvents^39^. In the case of the alkaline co-precipitation synthesis process, the nucleation is an unending phenomenon. This was observed by Baumgartner *et al*. with the help of real-time TEM. They reported that in such synthesis processes the nanostructures are formed due to the aggregation of the primary particles rather than atomic accretion, hence suggesting a secondary nucleation and growth mechanism^40^.

## Materials and Methods

**Figure 1:**
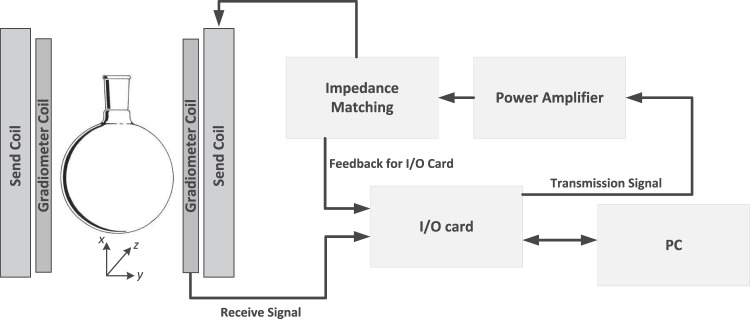
The signal chain of INSPECT. The signal for the transmitting coil is generated by an I/O card and amplified with a power amplifier. A gradiometer coil is used for acquiring the particle signal which is sent to the I/O card for analysis as well as displaying the data with a PC. The feedback to the I/O card is used for monitoring and controlling the voltage on the transmitting coil.

In this section, the hardware and software realization of INSPECT is described. The diameter of the measurement chamber is 72 mm and can fit a 100 ml reaction flask for synthesis (see Fig. 1). The complexity of the hardware modules is quite modest compared to that of recently described MPS systems^21–23^.

### Signal generation and acquisition

For the generation of the transmitted signal *T*_*x*_ and acquisition of the received signal *R*_*x*_, an X3-25M (Innovative Integration, USA) is used^41^. The X3-25M features two 16-bit ADC (analog to digital converter) channels and two 16-bit DAC (digital to analog converter) channels. Moreover, the ADC has programmable input ranges and a bandwidth of 75 MHz. The DAC channels have an output of ±2*V*. One of the DAC channel is used for generating the transmitted signal for the transmitting coil. The resulting voltage across the transmitting coil is fed back to the I/O card to control the flux density inside the measurement chamber. The X3-25M generates the sinusoidal *T*_*x*_ signal at 23 kHz with a sampling frequency of 5.06 MSPS (mega samples per second), ensuring a high signal quality. *R*_*x*_ is also acquired with the X3-25M at a sampling rate of 5.06 MHz. Both signals are synchronized with the help of the timing unit present on the I/O card itself. The total measurement time for one measurement is about 1 s acquiring 10 periods with 2300 averages. The same measurement parameters are used for analyzing the sensitivity of INSPECT as well as for monitoring the synthesis process described in results and discussion.

### AC power amplifier

**Figure 2:**
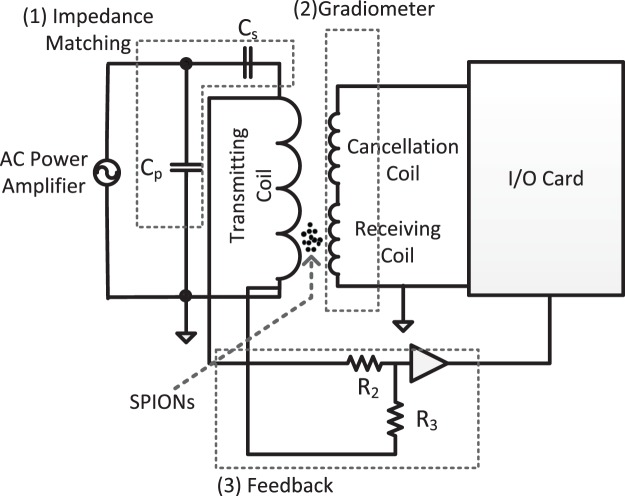
The electrical circuit diagram of INSPECT. The impedance matching unit (1) consists of two capacitors one in series C_s_ and the other one in parallel C_p_. The gradiometer coil (2) consists of the receiving coil and the cancellation coil. The feedback (3) consists of a voltage divider R_2_ and R_3_ and an operational amplifier for impedance matching the signal to the I/O card. The gradiometer coil is positioned such that there is no need for either a bandstop filter or a low noise amplifier to detect the signal from the SPIONs.

The *T*_*x*_ signal generated by the I/O card has to be amplified by a power amplifier (AE Techron, USA) to provide the necessary magnetic field of up to 10 mT^42^. This amplifier has a low total harmonic distortion (THD) of less than 0.1% at *f*_0_ = 23 kHz (fundamental frequency) with a maximum gain of 20. This reduces the need for analog filter stages, which are usually employed for the send chain in a conventional MPS setup. To ensure that the maximum power is transferred to the transmitting coil and to minimize the reflections between the AC power amplifier and the transmitting coil, a capacitive impedance matching is used. The circuit diagram for the impedance matching and the overall design is shown in Fig. 2. The feedback consists of a voltage divider and an amplifier to measure the changes in the magnetic flux density.

### Transmitting coil and gradiometer coil

**Figure 3:**
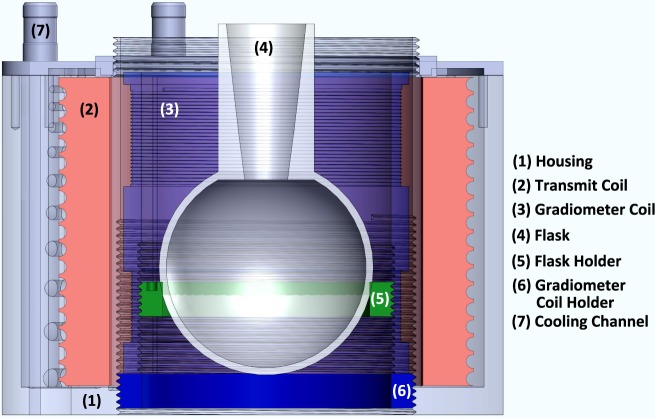
The field generator of INSPECT consisting of (1) the main body which houses the transmitting coil, (2) the transmitting coil, (3) the gradiometer coil, (4) the flask for synthesis, (5) the flask holder, (6) the gradiometer coil holder, which could be mechanically adjusted to attain the maximum sensitivity of the particle signal and attenuation of the excitation frequency, and (7) the cooling channel for pumping pressurized air in and around the transmitting coil and the gradiometer coil.

The transmitting coil is responsible for the generation of the required field to excite the SPIONs. The gradiometer coil cancels the transmitting signal out and provides the response of the nanoparticles for further analysis. The entire design of the transmitting coil and the gradiometer coil with the housing is shown in Fig. 3.

#### Transmitting coil

The transmitting coil generates a magnetic field and consists of a solenoid. The outer diameter of the transmitting coil is 121 mm and the inner diameter of the coil is 92 mm, which can easily fit a glass flask of 100 ml for synthesis. The height of the transmitting coil is 93.75 mm. The transmitting coil consists of 40 × 4 windings realized with 2000 × 5 μm Litz wire. The inductance of the coil is approximately 229 μH at 23 kHz (which is the excitation frequency of INSPECT). The air cooled transmitting coil is able to produce a field of up to 10 mT.

#### Gradiometer coil

The receiving coil lies inside the transmitting coil, which leads to higher mutual inductances causing a direct feed through of the excitation signal to the receiving coil. This excitation signal is usually 5–10 times higher than the nanoparticle signal and therefore has to be damped in the receiving channel. Most of the state of the art MPS devices rely on a compensation coil or bandstop filters to dampen the excitation signal in the receiving channel^22^. Other techniques have been employed such as a dedicated cancellation unit^21^ and perpendicular sensing^43^. A comparison between some of these techniques can be found in Graeser *et al*.^44^. For the MPS presented here, a one-dimensional gradiometer coil is used, which is a receiving coil including a cancellation coil (with reverse winding). Gradiometer coils have been used in a number of fields like for constructing magnetometers^45^, in geology^46^ as well as in medical science^47^. For fine tuning, the gradiometer coil holder, as well as the flask holder (shown in Fig. 3), can be mechanically adjusted to achieve the maximum attenuation of the excitation signal in the receiving channel. The gradiometer coil consists of pure copper wire with a diameter of 0.50 mm and has 30 turns in the receiving coil and approximately 28 windings in the cancellation coil and has a diameter of 74 mm. The CAD drawing of the entire setup is shown in Fig. 3.

### Housing

The housing for the transmission coil and the gradiometer coil is manufactured with a ProJet 3510 HDPlus 3D printer (3D Systems, USA). The printed material can withstand temperatures up to 80 °C. The cooling of the coils is realized by pressurized air. Through the cooling channel the air is channeled over the transmission as well as the receiving coil. For chemical reactions requiring higher temperatures such as organic synthesis, the housing can be manufactured with the help of high-temperature resins which can withstand temperatures of up to 238 °C. One such example of a material for additive manufacturing is from Formlabs (Formlabs Inc., USA) called High Temp Resin (FLHTAM02) offering a heat deflection temperature (HDT) of 238 °C at 0.45 MPa^48^.

### Calibration

As the signal could lose its fidelity while passing through the receiving chain or the sending chain, it is important to calibrate the system. As INSPECT has no filters or pre-amplifiers in either the sending chain or the receiving chain, the transfer functions of the chains are free of heating effects and can easily be determined.

#### Transmission chain calibration

For calibration of the transmitting chain, a transmitting calibration coil (*u*_Tcal_) made up of a copper wire and a voltage divider circuit is used. This coil is mounted on a cylinder of 51 mm in diameter with 21 windings of 0.40 mm pure copper wire. The transmitting calibration coil (*u*_Tcal_) detects the magnetic field generated by the transmitting coil. With the known geometry and the received voltage, the magnetic field can be easily calculated by using the Faraday’s law of induction. The applied magnetic field is given by

7 $${B}_{Tx}(t)=-\,\frac{{u}_{{\rm{Tcal}}}}{{f}_{0}\cdot A\cdot N}$$

where *B*_*Tx*_(*t*) is the generated magnetic flux density, *f*_0_ is the fundamental frequency of 23 kHz, *A* is the cross-section area, and *N* is the number of turns in the transmitting calibration coil. Using Eq. 7 the field strength and phase of the exciting magnetic field can be determined.

#### Receive chain calibration

**Figure 4:**
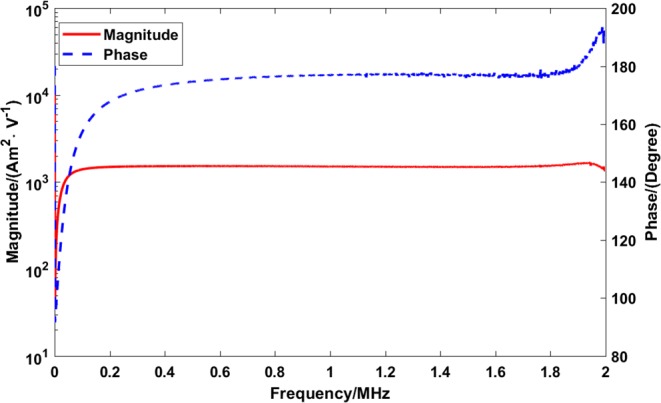
The transfer function of the receiving chain. The peak at approximately 2 MHz is the receiving coil self-resonance peak.

In general, components such as filters and amplifiers add distortions to the signal detected at the receive chain. To correct for these distortions, the final measurement has to be corrected by the transfer function of the receiving chain. In the current research the transfer function is obtained with a 20 mm receive-chain calibration coil made by pure copper wire of 0.5 mm thickness having 5 windings. The coil is connected to a 500 Ω series resistor to measure the current. The phase and magnitude of the measured transfer function are shown in Fig. 4. As the absence of filters and amplifiers in our receive chain implies, the transfer function is straight in a wide frequency range.

### SPION synthesis

For the synthesis of SPIONs there are a number of methods, but usually, in biomedical science, the three most investigated processes are alkaline precipitation in water, water-in-oil micro-emulsions and the thermal decomposition of organometallic iron in organic solvents^49^. For the validation of INSPECT, the alkaline co-precipitation in water is used, which is the most simple and common method, however leading to a broad core-size distribution. This process comprises of two stages, in the first stage there is a nucleation, which is followed by a slow growth in the second stage. The chemical reaction for the formation of the iron oxide can be divided into two steps:

8 $$2F{e}^{3+}+F{e}^{2+}+8O{H}^{-}\to Fe{(OH)}_{2}+2Fe{(OH)}_{3}$$

9 $$Fe{(OH)}_{2}+2Fe{(OH)}_{3}\to F{e}_{3}{O}_{4}+4{H}_{2}O$$

For the synthesis the iron salts, iron (III) (1.32 g of *FeCl*_3_ · 6*H*_2_*O*, ≥99% obtained, Carl Roth GmbH Karlsruhe, Germany) and iron (II) (0.50 g of *FeCl*_2_ · 4*H*_2_*O*, ≥99% obtained, Merck kGaA, Darmstadt, Germany) plus Dextran T70 (1.32 g, AppliChem GmbH, Darmstadt, Germany) are placed in a round bottle flask inside INSPECT and ammonia as a base is dropped into the solution. The flow rate of the base is controlled with an infusion pump (PERFUSOR secura FT, B. Brown), which is set to 20 ml/h. After the addition of the base, the mixture is slowly heated to a temperature around 80 °C under ultrasonic control^50^, where the growth of SPIONs takes place.

### Measurement protocol

Before initiating the synthesis process, an empty measurement is taken, which is subtracted from the subsequent measurements to correct the background noise. The second measurement is taken after putting the iron salts and dextran and water in the flask. Then, 25 ml of base (ammonia (*NH*_3_) for the current research) is dropped controlled by an infusion pump at 20 ml/hr. After the addition of all the required base, the mixture is slowly heated to approx. 80 °C for 100 min. Measurements are taken every 5 min over the entire duration of the synthesis process. For determining the sensitivity of INSPECT a 10 μL Resovist sample is used.

## Results and Discussion

In this section, initial results of synthesis monitoring with INSPECT are presented and discussed. The change in the magnetic properties during the synthesis is demonstrated. The measurements consist of both the amplitude and the phase spectra and the hysteresis curve at different stages of the synthesis. Hysteresis curves are derived by measuring the magnetization of the nanoparticles depending on the dynamic excitation field. The dynamic excitation field is monitored using the feedback signal consisting of the voltage divider as shown in Fig. 2. The minimum measurement time possible is the length of one period of the excitation field, which in this case is approximately 42.9 μs.

### Reference measurement for INSPECT

**Figure 5:**
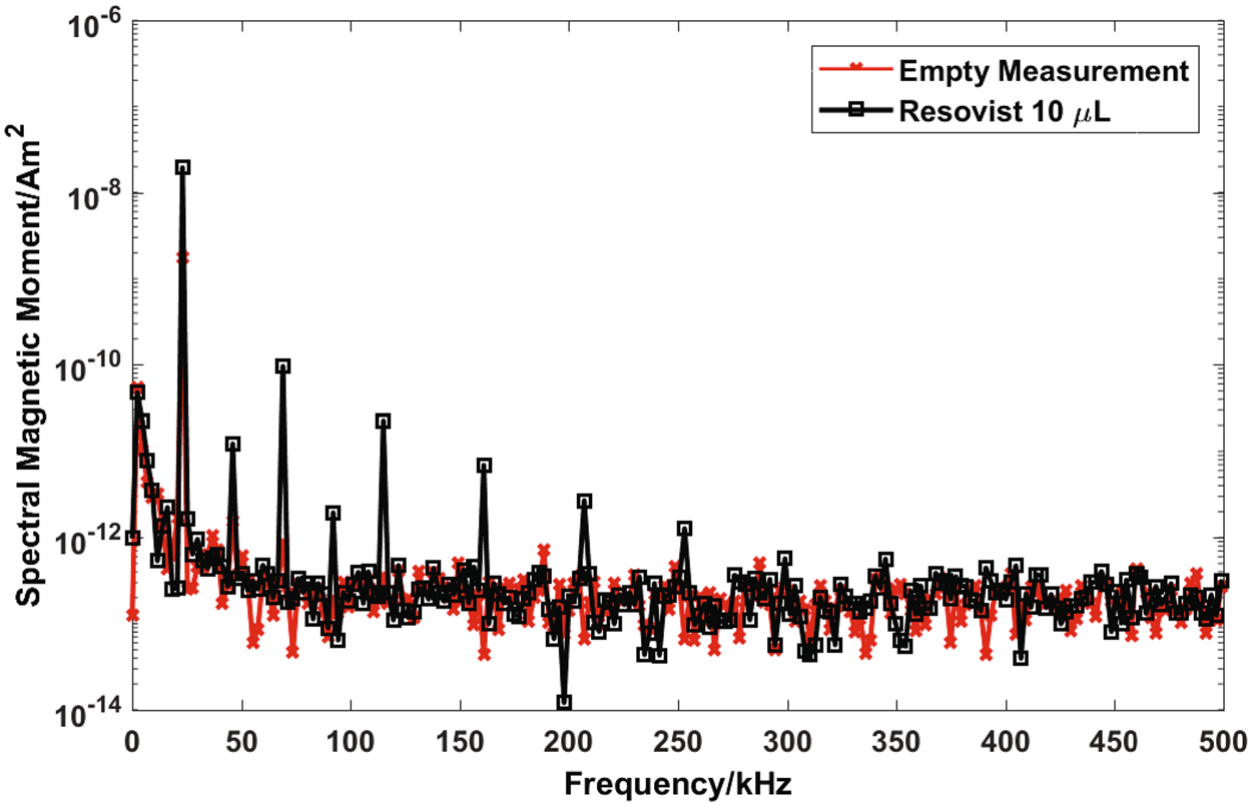
The empty measurement is shown in red and the spectral magnetic moment of a 10 μL sample of Resovist is shown in black. The sample was placed in the middle of INSPECT where the receiving coil of the gradiometer coil is located. The first six odd harmonics up to 253 kHz are clearly distinguishable from the noise floor.

INSPECT works very efficiently with a high concentration of SPIONs in suspension. However, it is crucial that the device can also detect small concentrations of SPIONs, because the SPIONs have a small size in the nucleation phase of the synthesis process. In this section, results of an experiment are presented that demonstrates the lowest quantity of SPIONs detectable with INSPECT. For this particular analysis, commercially available SPIONs (Resovist, Bayer, Berlin) are used. Resovist is a multicore particle with an iron oxide core consisting of multiple single crystals of approximately 4.2 nm in diameter^51^. The spectral response of the Resovist samples are compared to an empty measurement after subtracting the background. Figure 5 shows the background and the amplitude spectrum of Resovist. The empty measurement is shown in red and the signal of a 10 μL (containing 0.28 mg of iron) Resovist sample is shown in black. INSPECT is able to detect the first 6 odd harmonics. This ensures that the device is capable of detecting small quantities of SPIONs. Furthermore, even harmonics are also visible in the amplitude spectrum, this is due to the presence of a slight DC offset in the produced field.

### *In-situ* magnetic particle measurements during particle synthesis

**Figure 6:**
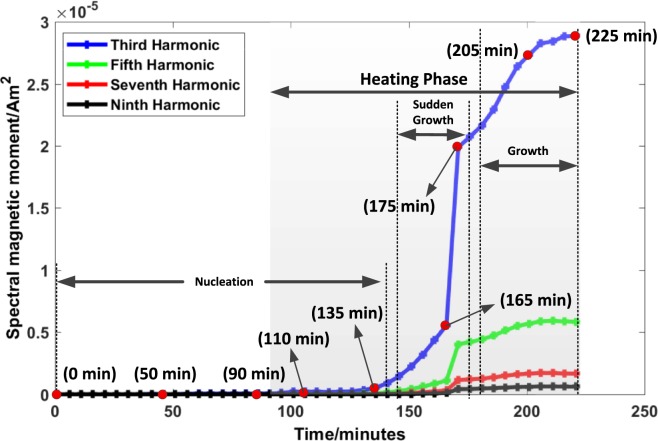
The magnetic moment of the third, fifth, seventh, and ninth harmonics versus time of the entire synthesis process. The synthesis process can be subdivided into three different intervals. The first interval is the nucleation phase, where only a slight change in the magnetic properties can be seen over time. This is followed by a sudden growth phase. The last interval is the growth phase, where a slow linear particle growth takes place which converges into a static behavior. The synthesis process is stopped in this phase. The measurements marked with red points will be discussed in the next figures in detail. The heating phase is also indicated, which marks the time period in which the solution is heated up to 80 °C. Before the heating phase, the base is dropped into the solution containing the iron salts and dextran.

Figure 6 shows the change of magnetic properties in the colloidal solution versus time of the entire synthesis process. Starting from third harmonic to ninth harmonic are plotted versus time. For simplification and without loss of generalization, the discussion of the results is limited to the temporal change of magnetic moment in the third harmonic as the third harmonic has the maximum power in comparison to subsequent harmonics.

This particular synthesis can be divided into three different intervals. The first interval consists of the nucleation process, in which, for a long period of time, there is no or negligible formation of superparamagnetic material. As stated by Baumgartner *et al*. secondary nucleation and growth mechanisms widely occur in the co-precipitation synthesis process^40^. Therefore, it is very difficult to estimate when the nucleation phase has completely ended and growth has started.

This interval ends with the initial particle formation, which shows a weak magnetic moment. In the second interval, a sudden growth of the nanoparticles occurs. In a span of 30 min the magnetic moment increases from 5.32 × 10^−7^ Am^2^ to 1.99 × 10^−5^ Am^2^, which is approximately a factor of 37.4. This happens because the nuclei that had already formed in the nucleation phase reach a reasonable size to show a magnetic moment. In the last interval, there is a very slow growth and a small change by a factor of 1.45 in the magnetic moment from 1.99 × 10^−5^ Am^2^ to 2.887 × 10^−5^ Am^2^. At this point, the synthesis process is stopped as no further changes in the magnetic properties occur. For this particular synthesis, 46 measurements are taken in the total time span of approximately 225 min. In Fig. 6 specific measurement points are indicated (marked with red dots) that will be further discussed in detail.

#### Nucleation phase

**Figure 7:**
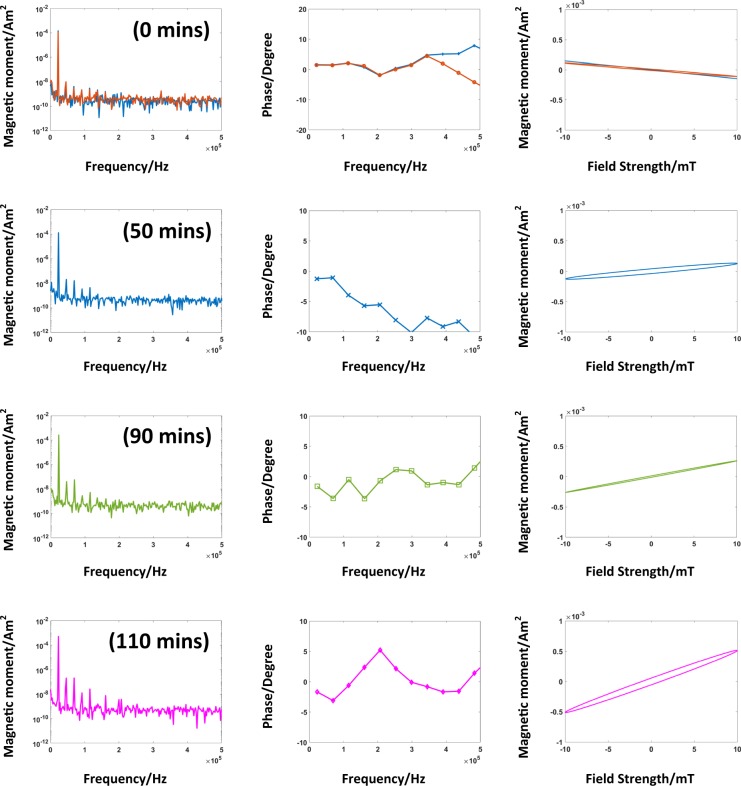
Measurements are taken in nucleation interval. The rows comprise the amplitude and phase spectra, and the magnetization curves at different time intervals. The first row, acquired at time stamp 0 min, includes two measurements, one is the empty measurement (marked in blue color) and the other one with the glass flask containing the reagents (iron salts, dextran) and water inside the chamber (marked in red color). The other measurements are performed at time stamp 50 min, 90 min, and 110 min, respectively. As all the above measurements have been acquired in the nucleation phase of the synthesis process, for the amplitude spectrum there are no significant changes till 90 min but at 110 min the fourth odd harmonic appears. There are significant changes in the phase spectra, and, in turn, in the resulting magnetization curves. Due to small amplitudes, the phase measurements are inaccurate and fluctuate. However, the area under the magnetization curve is maximum for the measurement at time stamp 110 min.

In the nucleation phase, there is no visible change in the magnetic properties of the nanoparticles as it takes some time for the solution to reach the supersaturation state to start the nucleation process. The nucleation occurs only when the concentration of the growth species increases above the equilibrium concentration, i.e. the concentration of the base increases in the solution. The base is being dropped slowly, therefore it takes around 80 min for completion. The slow addition of the base is important, because a sudden increase in the concentration of the base may lead to the formation of different sizes of nuclei and hence, leads to a broader size distribution^52^. Some of the measurements taken during this interval are shown in Fig. 7. The points at which these measurements are taken within the synthesis process are indicated in Fig. 6 (red dots with time stamps). Figure 7 shows the amplitude and phase spectra, and the magnetization curve at the different time points. The first time point comprises of two measurements in Fig. 7 at 0 min, the first one is an empty measurement (marked in blue). In the second measurement water and the flask containing all the reagents i.e. iron salts and dextran have been measured (marked in red). The diamagnetic behavior of water can be seen in the magnetization curve^53^. Followed by measurements after addition of base, which leads to the initialization of the nucleation. However, there are no significant changes in the magnetic properties observed, as supersaturation occurs locally, at the position where the base is being dropped. However, after a few seconds of ultrasonic stirring, the base is mixed into the solution. As shown at 0 min, there is a negligible change in the amplitude of the signal received, but there is a prominent change in the phase and the shape of magnetization curve for the subsequent measurements at 50 min. This process continues for measurements till 50 min and 90 min, leading to phase changes and shifting of the magnetization curve. In the measurements at 50 min and 90 min, respectively, it can be seen that the magnetization curve is changing. Moreover, the amplitude of the third harmonic increases for the measurement at the time stamp at 50 min to measurement at 90 min. In the nucleation phase, the detection of SPIONs is challenging as the magnetic moments of the particles are small.

#### Sudden growth phase

**Figure 8:**
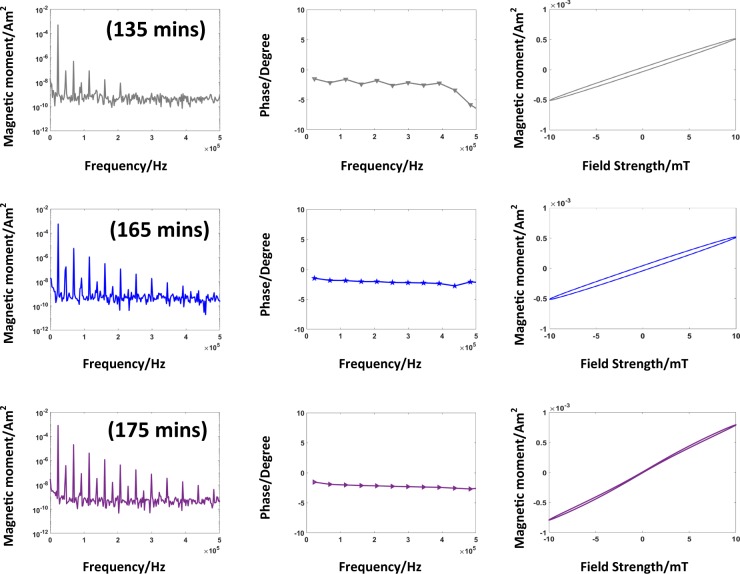
Sudden growth phase. The measurements comprise of the amplitude and phase spectra, and the magnetization curve, respectively. The first row is acquired when the growth is initiated at the time stamp at 135 min. In comparison to the measurements in Fig. 7, there is a significant increase in the amplitude of the odd harmonics. This directly implies that there is further growth in the particles, which are formed in the nucleation phase. The measurements at the time stamp 165 min and 175 min show harmonics up to 500 kHz in the spectral magnetic moments, which confirms the particle growth. At time stamp 175 min, the magnetization curve indicates the beginning of the saturation effect. Furthermore, with the progression of the synthesis the phase becomes much more stable. As it can be seen by comparing the phase spectra at time stamp 135 min to the time stamp at 175 min.

When the required quantity of the base is added to the solution, the heating is started. This leads to a decrease in the concentration of the growth species and the change in the volume Gibbs free energy decreases. Ideally, this should not lead to further nuclei formation and a coherent growth process continues until the concentration of growth species reaches the equilibrium concentration^39^. For alkaline based synthesis this is not the case. Figure 8 shows the measurements taken in this phase. These measurements are marked in Fig. 6 and are acquired at 135 min, 165 min, and 175 min, respectively. The measurement at the time stamp 135 min is acquired before the sudden growth initiates. The amplitude and phase spectra, and the magnetization curve obtained are shown in Fig. 8. The spectral magnetic moment indicates that superparamagnetic particles reached the targeted size in the solution, as the higher odd harmonics appear in the spectrum, which is a prerequisite for a good resolution for MPI^3^.

The next measurement is taken at 165 min. In the amplitude spectrum, the first nine odd harmonics can be observed and the phase spectrum shows linearity. The magnetization curve shows no saturation at field strengths of 10 mT. This situation changes in the following 10 min as shown in measurement at the time stamp at 175 min. There is a sudden growth of the particles, which significantly increases the magnetic moment. The magnetization curve also shows a saturation effect indicating larger particles and the phase spectrum is almost constant.

#### Growth phase

**Figure 9:**
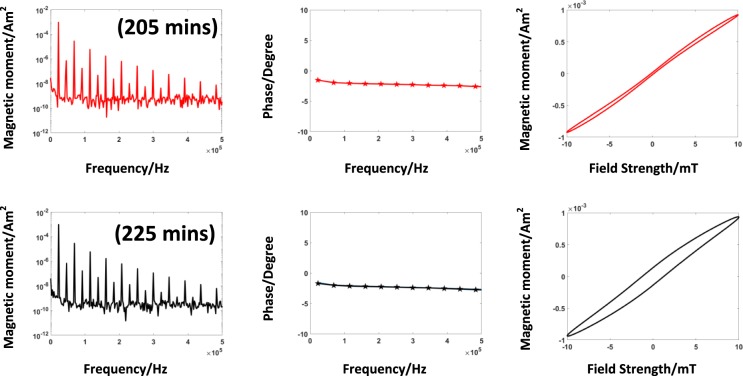
Growth phase. The first row shows the results of the data acquired at the time stamp at 205 min and the last measurements at 225 min, which finalizes the series. The hysteresis of the magnetization changes significantly. The phase spectra remain stable for both measurements and there is a negligible change in the amplitude spectrum.

The third interval is the growth phase, where the SPIONs show a linear growth behavior. The results are shown in Fig. 9. There is an increase in the area of the magnetization curve over time. The measurements have been acquired after 205 min and 225 min after the synthesis initiation. The measurement at the time stamp 225 min marks the termination of the synthesis process as no significant change in the magnetic properties over time can be observed.

## Conclusion

An *in-situ* MPS is presented, which is capable of tracking the growth of the nanoparticles in an ongoing synthesis process. The hardware realization is compact, consists of few hardware components, and is well suited for the chemical laboratory. The whole setup consists of a housing consisting of a transmitting coil and gradiometer coil. In addition, there is an impedance matching module and a power amplifier. There are no additional band pass or band stop filters for either the transmitting signal or the receiving signal. The excitation frequency can easily be changed by switching the capacitors in the impedance module. As the gradiometer coil is vertically movable, it can be adapted to receive the maximum signal and to cancel out the excitation signal.

The measurements presented here have been acquired every 5 min, however, the sampling rate is adaptable to real-time measurements with increasing cooling effort. To demonstrate the usefulness of the device a complete alkaline precipitation based synthesis process of SPIONs has been monitored with INSPECT. A series of data sets have been acquired over a time span of 225 min. From the results it can be deduced that the synthesis process can be divided in three main intervals, i.e. the nucleation phase, the sudden growth phase, and the growth phase. In the nucleation phase there is no significant change in the amplitude spectrum. In the nucleation phase, there is a decrease in the coercivity for the measurement at time stamp 90 min to 55 min. As in the nucleation phase there are a lot of competing processes happening such as particle coarsening (also called Ostwald ripening) and digestive ripening^54^. Ostwald ripening leads to dissolving of the smaller particles back to solution and a redeposition on the larger particles. On the other hand, digestive ripening leads to shirking of the larger particles leading to further growth of the smaller particles. These processes can cause changes in the Brownian relaxation and in turn affect the magnetic moment of the particles. With INSPECT we will be able to study these phenomena in more detail. In the sudden-growth interval, as the SPIONs become bigger in size, the spectral magnetic moment changes significantly. This phase ends in the growth interval, where particles grow slowly and finally due to no significant changes in the spectral magnetic moment, the synthesis process is stopped. INSPECT is able to monitor these changes and can be used to optimize the synthesis process, because it allows for an insight into the growth dynamics and reveals the direct effects of synthesis parameter tuning. This device provides a platform to study the dynamics of different synthesis processes to improve SPIONs for applications in both MRI and MPI.

## Data Availability

All data generated or analyzed during this study are included in this published article.
